# Malaria and leishmaniasis: Updates on co-infection

**DOI:** 10.3389/fimmu.2023.1122411

**Published:** 2023-02-21

**Authors:** Uyla Ornellas-Garcia, Patricia Cuervo, Flávia Lima Ribeiro-Gomes

**Affiliations:** ^1^ Laboratory of Malaria Research, Oswaldo Cruz Institute, Oswaldo Cruz Foundation, Rio de Janeiro, RJ, Brazil; ^2^ Malaria Research, Diagnosis and Training Center (CPD-Mal) of Oswaldo Cruz Foundation, Rio de Janeiro, RJ, Brazil; ^3^ Laboratory on Leishmaniasis Research, Oswaldo Cruz Institute, Oswaldo Cruz Foundation, Rio de Janeiro, RJ, Brazil; ^4^ Rio de Janeiro Research Network on Neuroinflammation, Oswaldo Cruz Institute, Oswaldo Cruz Foundation, Rio de Janeiro, RJ, Brazil

**Keywords:** co-infection, tropical diseases, *Plasmodium*, *Leishmania*, immune response

## Abstract

Malaria and leishmaniasis are endemic parasitic diseases in tropical and subtropical countries. Although the overlap of these diseases in the same host is frequently described, co-infection remains a neglected issue in the medical and scientific community. The complex relationship of concomitant infections with *Plasmodium* spp. and *Leishmania* spp. is highlighted in studies of natural and experimental co-infections, showing how this “dual” infection can exacerbate or suppress an effective immune response to these protozoa. Thus, a *Plasmodium* infection preceding or following *Leishmania* infection can impact the clinical course, accurate diagnosis, and management of leishmaniasis, and vice versa. The concept that in nature we are affected by concomitant infections reinforces the need to address the theme and ensure its due importance. In this review we explore and describe the studies available in the literature on *Plasmodium* spp. and *Leishmania* spp. co-infection, the scenarios, and the factors that may influence the course of these diseases.

## 1 Introduction

The risk of contracting an infection is inherent in the life of any living being. The same individual can encounter a variety of viruses, bacteria, helminths, fungi, or protozoa throughout a lifetime and often in the same period of time, increasing the chances of a co-infection (1, 2). Co-infection is defined as infection of the same host by two or more microorganisms belonging to different species or strains (1, 3).

The effect of co-infection on disease pathogenesis is related to the type of interaction that the parasites establish with each other and with the host, including the immune response triggered against these pathogens (3–5). In a co-infection, the parasites may cause a similar immune response profile (which may exacerbate this response) or a different one, bringing limitations to the immune response, with increased susceptibility and lack of control of the infection (6). Therefore, interactions between parasites that inhabit the same host simultaneously can be neutral, synergistic or antagonistic (1–3).

**Figure 1:**
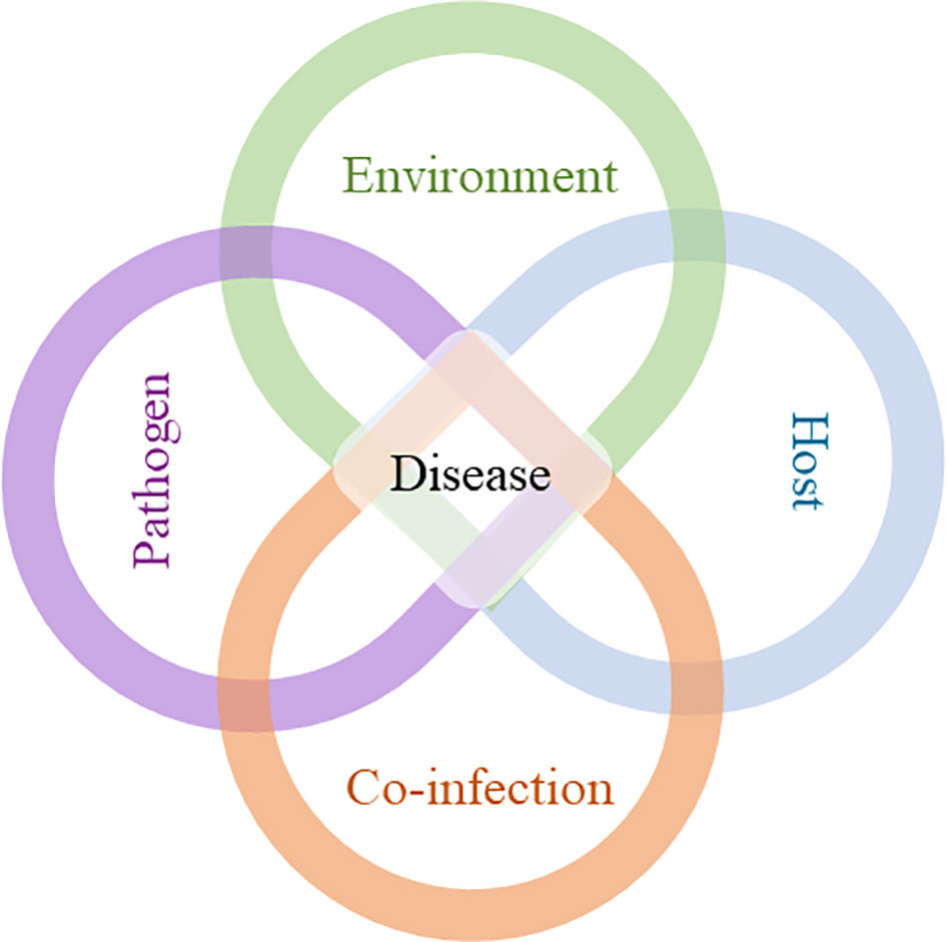
Development of a disease: interplay between environment, host, pathogen, and co-infection. The course of disease development cannot be sufficiently predicted by the interaction of host, pathogen and environment alone. Co-infection (s), along with the other factors, can interfere with the duration of infection, level of infectivity, clinical manifestations, and transmission, which can compromise disease control.

The order of arrival of pathogens in the host is another crucial factor to be considered. When we talk about co-infection, it is immediately assumed that at least two parasites infect the host simultaneously, but this only happens in specific cases, when a particular vector carries different pathogens and transmits them to the host at the same time (5). However, what is more feasible in nature is sequential co-infection, where the first parasite infects the host and then the second parasite, within a certain period (which can be from days to years), finds the same host and infects it (5). Therefore, at the time of co-infection, the host environment is metabolically and immunologically different compared to an uninfected host. Thus, the manifestation of a given disease is not only dependent on host susceptibility, pathogen virulence and environmental factors, but can also be directly influenced by co-infections (**Figure 1**).

The geographical overlap of malaria and leishmaniasis has been reported through the presence of the parasites and specific vectors circulating in the same endemic regions (7, 8), showing that there is a great potential for interaction between these pathogens in the same host. Indeed, human co-infection with *Plasmodium* and *Leishmania* parasites has been described in nature (9–27). Despite that, and the impact that the coexistence of the two parasites in the same host can have on the course of the diseases, there are few studies in the literature that address this interaction. The purpose of this review is to provide an overview of the knowledge available in the literature on co-infection by the parasites that cause malaria and leishmaniasis. Articles detailing this interaction permeate natural co-infection in humans and experimental models of co-infection (**Table 1**).

## 2 Malaria and leishmaniasis: An overview

Malaria is an infectious parasitic disease caused by protozoa of the genus *Plasmodium* and transmitted by the bite of the *Anopheles* mosquito (7). Despite being an ancient disease, it remains a major public health problem worldwide. According to the *World Malaria Report* (37), an estimated 247 million cases of malaria occurred in 2021, with 619,000 deaths. In 2020, there was a significant increase in the numbers of malaria deaths (620,000) compared to the previous year (568,000). Overall, between 2019 and 2021, an estimated 13.4 million additional cases of malaria and 63,000 deaths can largely be attributed to the disruption of malaria prevention, diagnosis, and treatment program during the COVID-19 pandemic (37–39). Human malaria is mainly caused by five species of *Plasmodium*: *P. falciparum*, *P. vivax*, *P. malariae* and *P. ovale curtisi* and *P. ovale wallikeri* (7, 40, 41). However, there are three other zoonotically transmitted species that are also capable of infecting humans: *P. knowlesi, P. cynomolgi* and *P. simium* (40–45).

*Plasmodium* species show differences in their geographic distribution, pathological severity, and biology. *P. falciparum* is the predominant species on the African continent and is found in other tropical and subtropical regions. It is considered the species with the greatest impact on public health due to the high morbidity and mortality rates associated with severe forms of the disease, in addition to drug resistance (46). *P. vivax* is the most widely distributed species in the world. This parasite is the leading cause of malaria outside Africa and is found in regions of Asia and the Americas (46, 47). In the early stages, malaria has nonspecific symptoms, such as fever, chills, sweating, headache, and muscle pain. This set of symptoms is observed in most patients, but some cases, the disease can progress to more severe manifestations, such as pulmonary malaria, severe anemia, and cerebral malaria.

According to the WHO guidelines, the recommended treatment for adults (excluding pregnant women in their first trimester) and children with uncomplicated malaria caused by *P. falciparum*, *P. vivax*, *P. ovale*, *P. malariae* or *P. knowlesi* is with artemisinin-based combination therapies (ACT). This can include artemether + lumefantrine, artesunate + amodiaquine, artesunate + sulfadoxine–pyrimethamine (SP), and others (48). Chloroquine can also be used to treat uncomplicated non-*P. falciparum* malaria in areas with chloroquine-susceptible infections. In specific cases, to prevent *P. vivax* or *P. ovale* relapse, treatment should be carried out with primaquine, paying attention to the risk of G6PD deficiency. For severe malaria, the treatment of choice is intravenous or intramuscular administration of artesunate, followed by combination ACT therapy (48).

Leishmaniasis is a group of neglected infectious diseases caused by protozoan parasites of the genus *Leishmania* and transmitted by the bite of infected sand flies. It is estimated that 700,000 to 1 million new cases occur annually, and 1 billion people live in endemic areas and are at risk of infection (8) Endemic in 98 countries and 3 territories (49), in tropical and subtropical regions and in the Mediterranean basin, leishmaniasis is associated with lack of financial resources, precarious housing, climate change, population displacement, malnutrition and compromised immune system (8, 50, 51). More than 50 species of *Leishmania* have been identified, distributed in regions of the Old World (Europe, Asia, and Africa) and the New World (Americas). Of these, 20 species are known to infect humans (50, 52).

The disease, leishmaniasis, comprise a wide spectrum of clinical manifestations grouped in three main forms: Visceral Leishmaniasis (VL), Cutaneous Leishmaniasis (CL) and Mucocutaneous Leishmaniasis (ML) (8, 50). The VL form, also called kala-azar, is the most severe form of leishmaniasis. In the Old World, it is associated with infection by *L. donovani* and *L. infantum*, and in the New World by *L. infantum.* It is characterized as a systemic infection, with symptoms that include irregular fever, fatigue, abdominal pain, weight loss, splenomegaly and hepatomegaly (51, 52). Without treatment, VL is fatal in 95% of cases (8, 52). The CL form is the most common form of the disease. In the Old World, CL was mainly caused by *L. major* and *L. tropica*, and in the New World by more than eight different species of the *L*. (*Leishmania*) and *L*. (*Viannia*) subgenera, including *L. (L.) amazonensis*, *L*. (*L*.) *mexicana*, *L.* (*V.*) *braziliensis*, *L*. (*V*.) *panamensis*, *L*. (*V*.) *peruviana* and *L.* (*V.*) *guyanensis* (51, 53). The clinical presentation of CL varies according to the infecting species, the genetic background of the host, and immune response triggered. Typically, the lesions occur in the region of the sand fly bite and present as a single non-ulcerative and painless papule. However, multiple lesions can also occur as well as ulcerating dry or wet lesions with raised edges. Cutaneous lesions can heal spontaneously or after therapeutic interventions, but others can persist and be refractory to the treatment (51, 52, 54, 55). Between 2-14% of patients with CL caused by parasites of the *L*. (*Viannia*) subgenus develop ML (52, 56). In ML, the parasite disseminates from the skin to the oral and nasal mucosa through the lymphatic and hematogenous system, with slow and progressive destruction of these tissues (51, 52, 54, 57).

During the last six decades, the different forms of have been treated with a reduced option of drugs, including pentavalent antimonials, paromomycin, miltefosine, pentamidine isethionate and amphotericin B (52, 58, 59). Monotherapy with pentavalent antimonial was the first-line treatment for CL and VL worldwide, but due to the emergence of resistance or failure during treatment with this drug, its use is no longer recommended in some regions of the world (59, 60). Oral miltefosine monotherapy is being implemented by more countries in the last decade, as well as combination regimens of antimonial, paromomycin, liposomal amphotericin B, amphotericin B deoxycholate, pentoxifylline and miltefosine, according to the disease form and particular conditions of pediatric and adult patients (52, 58, 59).

It is well known that genetic factors influence resistance or susceptibility to infectious diseases, including malaria and leishmaniasis. However, few genome-wide association studies (GWAS) have been conducted to identify human gene polymorphisms associated with these diseases. Some genetic variations, such as those found in the hemoglobin subunit beta (*HBB*), ABO blood group (*ABO*), ATPase plasma membrane Ca2+ transporting 4 (*ATP2B4*), glucose-6-phosphate dehydrogenase (*G6PD*) and CD40 ligand (*CD40LG*) loci, have been associated with attenuation of severe malaria (61). In addition, polymorphism in the erythrocyte Duffy antigen/receptor for chemokines (*DARC*) gene is associated with resistance to *P. vivax* malaria (62, 63). Specific TNF and DDX39B haplotypes have also been linked to increased susceptibility to *P. vivax* infections in Brazil (64, 65). *DDX39B* encodes an RNA helicase that has been described as a negative regulator for the expression of TNF and IL-6.

Regarding leishmaniasis, recently, a robust GWAS identified lysosomal associated membrane protein 3 (*LAMP3*), Syntaxin 7 (*STX7*), Keratin 80 (*KRT80*), IFNG antisense RNA 1 (*IFNG*-*AS1*), Cytokine receptor like factor 3 (*CRLF3*), and Serpin family B member 10 (*SERPINB10*) as plausible genetic risk factors for CL caused by *L. braziliensis* (66). This study suggested that lower levels of IFN-γ and TNF, regulated by *IFNG*-*AS1*, increase the risk of CL. For VL, GWAS analysis identified a polymorphic HLA-DR-DQ region within the major histocompatibility complex of immune-related genes as the single major genetic determinant of VL (67). Interestingly, this association was found in three independent cohorts from India and Brazil, and represented infections caused by different parasite species, including *L. donovani* and *L. infantum*. Sakthianandeswaren A, et al. (68) and Bharati, K (69), have also described in their revisions several gene variations and their relationships with the course of different forms of leishmaniasis (68, 69). Despite the mentioned studies on genetic polymorphisms and parasitic diseases, to date, there has been no description of the association of certain polymorphisms with resistance or susceptibility to *Plasmodium* and *Leishmania* co-infection. This remains a challenging field for future studies.

**Figure 2:**
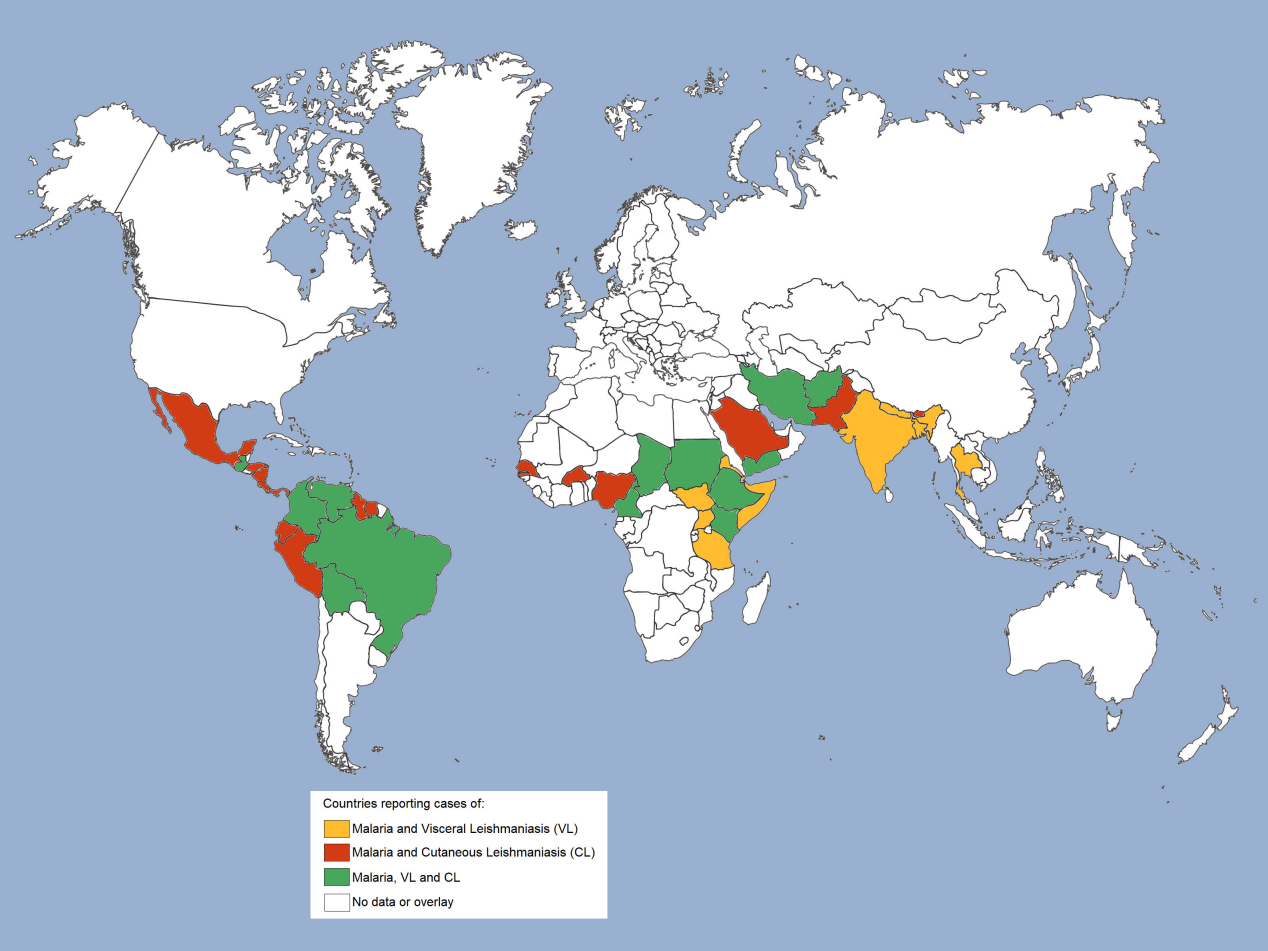
Geographical overlap of malaria and leishmaniasis cases worldwide. Geographical distribution of countries co-endemic for malaria and leishmaniasis. Cases of malaria and visceral leishmaniasis (VL) are reported in countries highlighted in mustard; cases of malaria and cutaneous leishmaniasis (CL) are reported in countries highlighted in red, and cases of malaria and both leishmaniasis (VL and CL) are reported in countries highlighted in green.

The geographical distribution of countries with reported malaria cases (39) or VL and CL cases in the world (70) reveals an overlapping of malaria, VL and/or CL cases in the Americas (Central and South), Africa and Asia, and suggests that at least 38 countries are at risk of co-infection (**Figure 2**). In brief, overlapping cases of malaria and CL are reported in Central America in countries such as Mexico, Costa Rica, Panama, Honduras, and Nicaragua, and in South America in Ecuador, Peru, Guyana, and Suriname. In addition, cases of malaria, CL and VL are observed in Guatemala, Colombia, Venezuela, Bolivia and Brazil (39, 56, 70, 71). On the African continent, cases of malaria and CL overlap mainly in the north of the continent, in countries with a predominantly tropical climate, such as Senegal, Burkina Faso and Nigeria. Also, countries in northeastern Africa, such as Somalia, Uganda, Djibouti, Tanzania, Eritrea, and South Sudan, have reported cases of malaria and VL (39, 70). And yet, cases of malaria, CL, and VL have been reported in Cameroon, Chad, Sudan, Ethiopia, and Kenya. In West Asia, malaria and CL are endemic in Saudi Arabia, while all three diseases are present in Yemen. In South Asia, cases of malaria and CL are found in Pakistan, and cases of malaria, CL and VL are found in Iran and Afghanistan (39, 71, 72). Malaria and VL cases have been reported only in India, Nepal, Bangladesh and Thailand (39, 70), while malaria and CL cases have been reported in Bhutan, a country located between China and India. Isolated cases of malaria or leishmaniasis are observed in several other countries, but without reports of co-endemicity.

## 3 *Plasmodium* spp. and *Leishmania* spp. co-infections

### 3.1 Natural co-infections

Seminal reports addressing natural co-infection by the parasites that cause malaria and leishmaniasis are from the 1930 and 1940 decades. To our knowledge, the first report of natural co-infection by *Plasmodium* spp. and *Leishmania* spp. in areas endemic for both parasites was made by Henderson et al. (9). Conducted on 300 patients with VL from a province in Sudan between 1933 and 1936, this study found the occurrence of concomitant infection with *Plasmodium* spp. in 30% of patients with VL (9). A few years later, Yoeli (10) observed the presence of non-pigmented malaria parasites, with characteristics similar to *P. vivax* in the bone marrow of a patient hospitalized with VL in Athens (10).

Since then, natural co-infection has been described in different regions of the world involving different *Plasmodium* and *Leishmania* species (**Table 1**). When assessing the concomitance of other infections in patients with leishmaniasis, *Plasmodium* was responsible for 10.7% of co-infection cases during a VL epidemic in southern Sudan in 1988 (20). *Plasmodium* co-infection was also described in 9,3% of VL cases in eastern Sudan in a retrospective study conducted from January 2013 to June 2014 (12) and, in 6.4% of VL cases in Pokot territory, located in western Kenya and eastern Uganda, a few years earlier, in 2006 (23). However, two other studies also conducted in Pokot territory, between 2000-2006, with a larger number of study subjects, describe a higher co-infection rate of 19-20.8% among patients with VL (24, 26). In Ethiopia, a study conducted between 2013 and 2018 showed that out of a total of 434 VL cases, *Plasmodium* co-infection is seen in 6.4% of cases in the Oromia region (17). Another study conducted in 2014 in northwest Ethiopia, on the border with Sudan, showed that 4.2% of VL patients were co-infected with *Plasmodium* (13). Overall, a meta-analysis study with data from 1991 to 2020 suggests that the prevalence of *Plasmodium* co-infection among individuals with VL ranges from 7 to 18% in Africa and Asia, according to the endemicity of VL and malaria in the evaluated area (18).

**Table 1:** Cases and studies on *Plasmodium* spp. and *Leishmania* spp.

	Malaria parasites	Leishmaniasis parasites	Natural or Experimental co-infections	Reference
**(a)**	*Plasmodium* spp.	*L. donovani*	Human	(9)
	*P. vivax*	*L. donovani* complex	Human	(10)
	*Plasmodium* spp.	*L. donovani*	Human	(20)
	*Plasmodium* spp.	*L. donovani*	Human	(21)
	*Plasmodium* spp.	*L. donovani*	Human	(22)
	*P. falciparum*	*L. donovani*	Human	(23)
	*Plasmodium* spp.	*L. donovani* complex	Human	(24)
	*P. vivax*	*L. donovani*	Human	(25)
	*P. falciparum*	*L. donovani*	Human	(26)
	*P. falciparum/P. vivax*	*L. donovani*	Human	(27)
	*P. falciparum*	*L. donovani*	Human	(11)
	*Plasmodium* spp.	*L. donovani*	Human	(12)
	*P. falciparum/P. vivax*	*L. donovani*	Human	(13)
	*P. falciparum/P. vivax*	*L. donovani*	Human	(16)
	*P. vivax*	*L. donovani*	Human	(14)
	*P. vivax*	*L. donovani*	Human	(15)
	*Plasmodium* spp.	*L. donovani*	Human	(17)
**(b)**	*P. berghei*	*L. infantum*	Golden hamster	(28)
	*P. yoelii* *P. berghei*	*L. enriettii*	Syrian hamster	(29)
	*P. yoelii*	*L. amazonensis*	BALB/c	(30)
	*P. yoelii*	*L. amazonensis*	C57BL/6	(31)
	*P. yoelii*	*L. amazonensis*	C57BL/6	(32)
	*P. yoelli*	*L. amazonensis*	BALB/c	(33)
	*P. chabaudi* AS	*L. infantum*	C57BL/6	(34)
	*P. falciparum*	*L. donovani*	*In vitro* (Dendritic cell culture)	(35)
	*P. yoelii* 17XNL	*L. amazonensis*/ *L. braziliensis*	BALB/c	(36)

Co-infection: (a) natural and (b) experimental (animal and *in vitro* model) co-infection.

To assess the prevalence and risk factors of co-infection by *Leishmania* and *Plasmodium* parasites, van den Bogaart et al. (26) followed patients admitted to a hospital in Uganda between 2000 and 2006. As mentioned above, of the 2,414 VL patients, 19% were diagnosed with malaria (26). Most of the co-infected patients were male, and age was considered a risk factor of co-infection, since the highest incidence of concurrent VL and malaria was observed in children of 0 to 9 years old. Children in this age group are twice as likely to be co-infected as adults over the age of 30 (26). The same group of investigators also conducted a study in three hospitals in Sudan between the years 2005-2010 evaluating malaria parasite co-infection in VL-hospitalized patients. Of the 1,295 VL patients, 31.2% were co-diagnosed with malaria (mainly caused by *P. falciparum*) at the time of hospital admission or during hospitalization (27), corroborating the prevalence of *Leishmania* and *Plasmodium* co-infection reported previously in the same region (22). Gender and age were considered risk factors for co-infection (27). The findings by Ferede et al. (13) also indicated that age was associated with co-infection by the parasites that cause malaria and leishmaniasis. They noted that the diagnosis of both diseases was significantly higher (33%) in children under 5 years old, who would possibly be more prone to co-infection because they have a less robust immune system (13). The second age group with the highest cases of co-infection was patients aged 15-29 years, where a 4% prevalence of dual diagnosis of VL and malaria was observed (13). Focusing on migrant workers, mostly male and aged between 15 and 29 years, Aschale et al. (16) found that the prevalence of co-infection among workers was 2.8% (16).

The concomitant occurrence of both infections in the same individual has implications for the clinical course of malaria and leishmaniasis. In a study conducted in East Africa, malaria is cited as a risk factor for the development of VL (23). The opposite, however, has not been thoroughly investigated. Although no differences in the prognosis of VL have been observed (as long as the *Plasmodium* infection is identified and treated effectively), co-infected patients have greater morbidity evidenced by loss of body mass, jaundice, and malaise (26, 27). Interestingly, an increased *Leishmania* parasite load was observed in lymph nodes or bone marrow aspirates in co-infected patients, suggesting a potential suppressive effect exerted by malaria parasites on *Leishmania* infection (27). On the other hand, reduced levels of *P. falciparum* parasitemia were detected in the co-infected patients compared to malaria-only patients, suggesting a protective effect of co-infection on *Plasmodium* infection (11). This effect may be related to the increased systemic levels of pro-inflammatory cytokines (TNF-α and IFN-γ) observed in the co-infected group when compared to patients infected with *Plasmodium* alone (11). The data indicate that the co-occurrence of the two diseases in the same individual interferes with the host’s immune response.

Co-infection cases have also been reported in India, accounting for 5.9% of patients with splenomegaly and fever (21). Interestingly, all patients diagnosed with VL, and malaria had symptoms that could be related to both diseases, such as fever, splenomegaly and hepatomegaly (15, 21). In a region of Nepal bordering India, another case of co-infection was reported, this time in a 5-year-old child, infected with *P. vivax* and *L. donovani*, with a history of fever, abdominal pain, constipation, and hepatomegaly (14). The data suggest that the clinical symptoms of co-infected patients hinder accurate diagnosis and, consequently, the initiation of appropriate treatment . Thus, delay in the recommended treatment may compromise treatment success and the course of the diseases (13, 15, 18, 25). However, co-infection is not reported to affect the efficacy of the recommended treatment for malaria or leishmaniasis if it is done in a timely manner.

Traditionally, we look for cases of co-infections with pathogens endemic to the same geographic area, but with the intense migratory activities and war refugees, this condition no longer applies. There may be imported cases of a particular infection, which is not endemic to that region. In an interesting case report, a patient from Nepal, a VL endemic area, while going to work in Malaysia (a malaria endemic area) tested positive for *P. vivax* two weeks after his arrival in the country. But even after complete treatment for malaria, the patient still had high fever, lack of appetite, splenomegaly, hepatomegaly, and pancytopenia. When the bone marrow aspirate was evaluated, amastigotes forms of *Leishmania* were found inside macrophages, showing that this was a case of co-infection by *Leishmania* and *Plasmodium*. This situation is considered challenging, since the patient probably contracted the *L. donovani* infection first at a different location and the *Plasmodium* infection, contracted later, interfered with the VL diagnosis, which could have been fatal (25).

### 3.2 Experimental co-infection: *In vivo* and *in vitro* models

Experimental animal models are valuable tools to investigate the pathological mechanisms of co-infections. The first study carried out to understand the dynamics of co-infection used Syrian golden hamsters (*Mesocricetus auratus*) inoculated with *L. infantum* and *P. berghei*. In this study, the authors observed that previous infection with *L. infantum* inhibited the multiplication of malaria parasites. On the other hand, an infection already initiated by *P. berghei* did not inhibit the multiplication of *L. infantum* (28). Using the same rodent model, the effect of *P. yoelii* infection on the course of infection by *L. enriettii*, a natural parasite of guinea pigs, was also evaluated. In contrast to what was observed by Adler et al. (28), when both infections were performed simultaneously or when *P. yoelii* was inoculated few days after *L. enriettii* infection, there was an increase in skin lesions in these two groups of co-infected animals when compared to the *L. enriettii* mono-infected group (29).

Like hamsters, murine models are excellent experimental models, being susceptible or resistant to different strains of *Plasmodium* spp. and *Leishmania* spp. according to their genetic background. Coleman et al. in their first studies on multiple infections, evaluated the co-infection by *P. yoelii* 17x (a non-lethal strain) and *L. amazonensis* in BALB/c and C57BL/6 mice, murine models considered susceptible and resistant to *L. amazonensis* infection, respectively (30–33). BALB/c mice infected first with *L. amazonensis* and a few days later (2 days, 3 days or 3 weeks) infected with *P. yoelii* 17x showed worsening of the skin lesion and *Plasmodium* infection with high parasitemia, marked hypothermia and elevated mortality rate (30, 33, 36). Similarly, C57BL/6 mice, when initially inoculated with *L. amazonensis* and subsequently (2 days or 3 weeks later) infected with *P. yoelii*, also showed increased footpad lesion and greater *P. yoelli* parasitemia compared to mono-infected animals (31, 33). Interestingly, in some co-infected animals, dissemination of the *L. amazonensis* was observed, with the appearance of a lesion in the contralateral uninfected paw, suggesting that concomitant infection with *Plasmodium* would have a potential to suppress the immune response, increasing the severity of cutaneous leishmaniasis in this model (32).

The impact of *P. yoelii* 17XNL infection on BALB/c mice parasitized by *L. braziliensis* was different from that observed in co-infection with *L. amazonensis* and *P. yoelli* (36). Animals co-infected with *L. braziliensis* and *P. yoelii* had lower parasitemia and smaller and less ulcerative lesions compared to mono-infected groups (36). Finally, a preliminary study in C57BL/6 mice infected first with *P. chabaudi* and then with *L. infantum* describes a higher susceptibility to leishmaniasis in co-infected animals. Co-infection, on the other hand, did not interfere with the course of malaria (34).

The effect of co-occurrence of the two infections in the same host on the elicited immune response was evaluated by Pinna et al. (36). Co-infection of BALB/c mice with *L. amazonensis* and *P. yoelii* or *L. braziliensis* and *P. yoelii* reduced serum levels of the cytokines IFN-γ, TNF-α, IL-6 and IL-10 induced by the malaria parasite. The data suggest a modulating effect of *Leishmania* infection on the immune response triggered by the *Plasmodium* parasite (36). Note that in malaria, the optimal immune response capable of controlling the parasite and causing no harm to the host involves an initial pro-inflammatory type 1 response, which later switches to a more regulatory response (73). Reduced serum levels of IL-10, an important regulatory cytokine, may have triggered an imbalance in the immune response and the worsening of malaria (74–77), contributing to the higher morbidity observed in animals infected with *L. amazonensis* and *P. yoelii.* From the standpoint of *Leishmania* infection, co-infection with *P. yoelii* increased serum levels of pro-inflammatory cytokines compared to animals infected with *Leishmania* alone. This immune response profile may have contributed to the reduced severity of *Leishmania* lesions observed in co-infected animals (36).

In summary, although the data suggest that co-infection shapes the susceptibility of the host to one or other infection, there is a considerable difference between the results obtained by the experimental co-infection studies. These differences are possibly related to the different species and/or strains of parasites involved in the studies, the genetic background of the host, and the time and order in which the infections occurred. All of these factors can dictate the clinical course and severity of the diseases (28–30, 33, 34, 36).

Similar to the *in vivo* experimental models, the *in vitro* co-culture system also provides important clues to understand how the immune response to a pathogen is triggered. Thus, it is quite common to use the *in vitro* co-culture system to assess how a specific parasite interacts with a particular cell type and modulates its function. In this regard, several studies have shown that exposure to *Plasmodium*-infected erythrocytes or hemozoin, a product of hemoglobin metabolism, negatively interferes with the antigen-presenting function of dendritic cells (DCs) (78–84). This negative impact on DC activation and maturation is also described in studies evaluating the interaction of the promastigote form of *Leishmania* with DCs (85–92). On the other hand, uptake of amastigote forms of *Leishmania* by DC induces its maturation and activation of T cells to a Th1 profile (92, 93). However, only one study so far has evaluated what happens to DC when interacting with both parasites, *Plasmodium* and *Leishmania*, simultaneously. In this study, monocyte-derived DCs (obtained from healthy donors) and co-stimulated with different concentrations of *L. donovani* promastigotes forms and *P. falciparum*-parasitized erythrocytes developed a semi-mature to immature profile, as the parasite/cell ratio increased, similar to DCs cultured with promastigotes forms of *L. donovani* alone (35). Suggesting a dominant effect of the *Leishmania* parasite-induced stimulus, over *Plasmodium*-induced stimulus, on the activation profile of DCs.

## 4 Conclusion

The geographical overlapping of the *Plasmodium* and *Leishmania* parasites, their vectors, and cases of natural co-infection are more than proven. Studies on natural and experimental co-infection, discussed in this review, suggest that the co-occurrence of the two parasites in the same host interferes with the duration of infection, level of infectivity and/or clinical manifestations. Some of these factors can influence the transmission and compromise the control of one or both diseases. This review also evidences the dangers of co-infection for populations residing in areas where these diseases are co-endemic. As the search for co-infections is not usually performed in these regions, the diagnosis of one infection may compromise the accurate and timely diagnosis of the other, implying a delay in treatment and possibly leading to patient death. Thus, more efforts should be made in an attempt to reduce the lack of knowledge about concomitant infections, and encouragement for the creation of programs that seek to diagnose co-infections by *Plasmodium* spp. and *Leishmania* spp. in areas co-endemic for both diseases.

## Author contributions

UO-G drafted the manuscript. UO-G, PC, and FL reviewed, edited, and prepared its final version. All authors contributed to the article and approved the submitted version.
